# The First Mitochondrial Genome for the Fishfly Subfamily Chauliodinae and Implications for the Higher Phylogeny of Megaloptera

**DOI:** 10.1371/journal.pone.0047302

**Published:** 2012-10-09

**Authors:** Yuyu Wang, Xingyue Liu, Shaun L. Winterton, Ding Yang

**Affiliations:** 1 Department of Entomology, China Agricultural University, Beijing, China; 2 Plant Pest Diagnostics Branch, Sacramento, California, United States of America; Onderstepoort Veterinary Institute, South Africa

## Abstract

Megaloptera are a basal holometabolous insect order with larvae exclusively predacious and aquatic. The evolutionary history of Megaloptera attracts great interest because of its antiquity and important systematic status in Holometabola. However, due to the difficulties identifying morphological apomorphies for the group, controversial hypotheses on the monophyly and higher phylogeny of Megaloptera have been proposed. Herein, we describe the complete mitochondrial (mt) genome of a fishfly species, *Neochauliodes punctatolosus* Liu & Yang, 2006, representing the first mt genome of the subfamily Chauliodinae. A phylogenomic analysis was carried out based on the mt genomic sequences of 13 mt protein-coding genes (PCGs) and two rRNA genes of nine Neuropterida species, comprising all three orders of Neuropterida and all families and subfamilies of Megaloptera. Both maximum likelihood and Bayesian inference analyses highly support the monophyly of Megaloptera, which was recovered as the sister of Neuroptera. Within Megaloptera, the sister relationship between Corydalinae and Chauliodinae was corroborated. The divergence time estimation suggests that stem lineage of Neuropterida and Coleoptera separated in the Early Permian. The interordinal divergence within Neuropterida might have occurred in the Late Permian.

## Introduction

Mitochondria are important functional organelles in eukaryotic cells [1], and the mitochondrial genome is being widely used for studies on evolutionary biology, because the mt genome sequences can be more phylogenetically informative than shorter sequences of individual genes, and provide multiple genome-level characteristics, such as the relative position of different genes, RNA secondary structures, and modes of control of replication and transcription [2]–[5]. Hitherto, in many mt genomic papers, while a well resolved topology is recovered, it frequently contradicts all previous estimates of phylogeny based on single sequences, nuclear genes, and even morphology [6]–[8], which might be caused by overly complicated evolutionary models among the mitochondrial genes, errors in methodology processing the genomic data, and biases in taxon sampling [9], [10]. As of 26 May 2012, 2627 complete Metazoa mt genomes have been sequenced and deposited in GenBank (http://www.ncbi.nlm.nih.gov), including 278 complete insect mt genomes from representative taxa of 26 orders. There are still four insect orders (i.e. Dermaptera, Zoraptera, Siphonaptera, and Trichoptera) with their mt genomes not yet reported.

**Figure 1:**
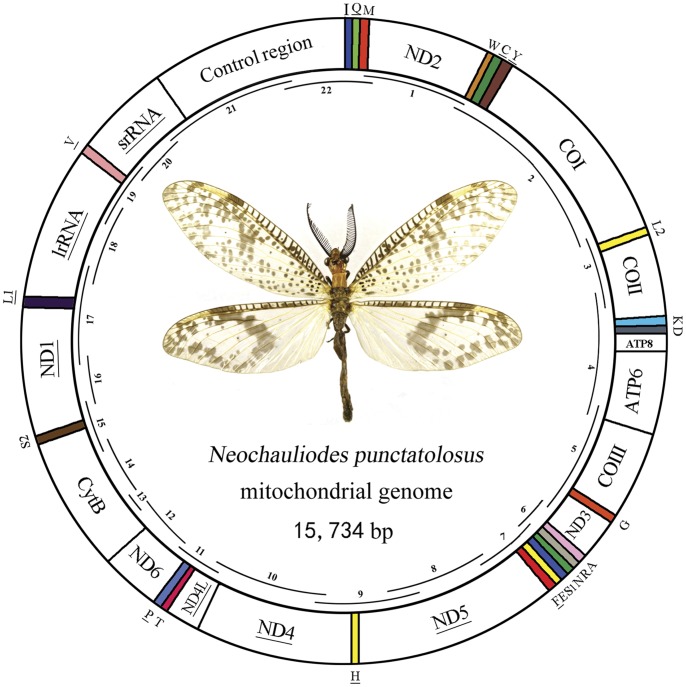
Mitochondrial map of *Neochauliodes punctatolosus*. The tRNAs are denoted by the color blocks and are labelled according to the IUPACIUB single-letter amino acid codes. Gene name without underline indicates the direction of transcription from left to right, and with underline indicates right to left. Overlapping lines within the circle denote PCR fragments amplified used for cloning and sequencing.

Megaloptera are one of the orders of the superorder Neuropterida (lacewings and allies) and are generally considered to be among the most archaic holometabolous insects because of their origin indicated by the earliest fossil evidence found in Late Permian (∼250 MYA) [11]. Megaloptera currently contain ca. 350 extant described species placed in two families, Corydalidae (dobsonflies and fishflies) and Sialidae (alderflies), both being widely distributed in all the zoogeographical realms, but with a large number of relic taxa remaining in the Southern Hemisphere. Adult Corydalidae are impressive and often look aggressive due to the large body (body length frequently greater than 90 mm) and wings, sometimes with distinctive colour patterns, and the tapered mandibles (Figure 1). Adult alderflies are generally diminutive (body length 5–15 mm) with subdued coloration. The larvae of Megaloptera are exclusively aquatic, predatory, and frequently dominate the predatory guild in lotic habitats such as streams, shallow rivers, ponds, etc [12].

Fossils of Corydalidae and Sialidae described from Early-Middle Jurassic deposits are morphologically very similar to extant relatives, indicating that this group has since undergone an relatively limited degree of morphological diversification [13], [14]. Due to morphological conservatism and consequent difficulties in identifying specific morphological apomorphies for the order in a phylogenetic context, the monophyly and higher phylogeny of Megaloptera has remained controversial [15], [16]. This is despite the monophyly of Megaloptera supported on morphological grounds by the presence of lateral abdominal tracheal gills in larvae, the position of the male ninth gonocoxite close to the base of ninth tergum and the eversible sacks of the male eleventh gonocoxites [17]–[19]. Additional characters of the larval head were defined by Beutel and Friedrich [20]. In contrast, a paraphyletic Megaloptera (i.e. Sialidae as sister to Raphidioptera) has been proposed repeatedly based on various lines of evidence including similarity of proximal fusion of M and CuA veins in the forewing [21], and shared specialization of telotrophic ovarioles [22]–[25].

The internal hierarchy of an assumed monophyletic Megaloptera has also been re-examined with an alternative hypotheses of cladogenesis recently proposed by Contreras-Ramos [15]. The traditional view holds that Sialidae are sister to Corydalidae, with Corydalidae further divided into two subfamilies (Corydalinae and Chauliodinae) [26]. Contreras-Ramos proposed that Corydalinae were sister to Chauliodinae + Sialidae based on morphological data [15].

The phylogenetic placement of Megaloptera as the sister group of Neuroptera is becoming stable through recent phylogenetic studies based on both morphological and molecular data [18], [19], [27], even though the traditional viewpoint that Megaloptera and Raphidioptera forms a monophyletic group was occasionally supported by recent studies of Holometabola relationships using substantial amounts of DNA sequence data from ribosomal [28] and nuclear genes [29], [30].

Recent studies on the molecular systematics of Neuropterida also generated controversial results regarding the higher phylogeny of Megaloptera. The first molecular phylogeny of Neuropterida inferred from four gene fragments suggested that Megaloptera as well as Corydalidae is monophyletic [31]. The monophyly of Megaloptera was also recovered in a phylogeny of holometabolous insects based on mt genomes [32]. Nevertheless, the latest comprehensive study on the molecular phylogeny of Neuropterida found that Megaloptera was not monophyly with Corydalidae to be the sister group of Raphidioptera [33]. To date, three mt genomes of Megaloptera have been determined for two dobsonfly species (*Corydalus cornutus* (L.) and *Protohermes concolorus* Yang & Yang) and one alderfly species (*Sialis hamata* Ross) [27], [34], [35]. However, the mt genome has not been determined for any fishfly species, therefore, all published phylogenies based on mt genomes of Megaloptera cannot clarify the relationships among the three main groups of this order [27], [32], [35]. With recent studies published on detailed morphological structure analysis [20] and large scale sequencing [30], [33], our understanding of the interordinal relationships of insects is becoming more complete [36]. In the holometabolous insects, Trautwein *et al*. identified only two clades where our understanding interordinal relationships is not supported by multiple sources of data, both within the clade Neuropteroidea [36]. These issues include in turn, the placement of Strepsiptera relative to Coleoptera and the rest of Neuropteroidea, and second, the monophyly and placement of Megaloptera relative to the rest of Neuropterida.

In this paper, we present the complete mt genome of a fishfly species, *Neochauliodes punctatolosus* [37], representing the first species from the subfamily Chauliodinae with the entire mt genome sequenced. We compared the genomic structure and composition, such as gene content, RNA secondary structure, and gene order, with other Neuropterida species (three species of Megaloptera, six species of Neuroptera, and one species of Raphidioptera) with their mt genomes already published [27], [34], [35], [38], [39]. A mt genome phylogeny comprising all three main groups of Megaloptera and all other Neuropterida families with available mt genomes is reconstructed for the first time based on the sequences of the entire set of protein coding genes (PCGs) and two rRNA genes. In addition, we estimated the divergence times with a relaxed-clock model of among-lineage rate evolution, aiming to present a timescale for the origin and diversification of Megaloptera. The results provide new evidence for the historical evolution of Megaloptera as well as the higher phylogeny of Neuropterida, and shed new light on the molecular timing of insects based on mt genome sequences.

## Results and Discussion

### Genome Organization

**Figure 2:**
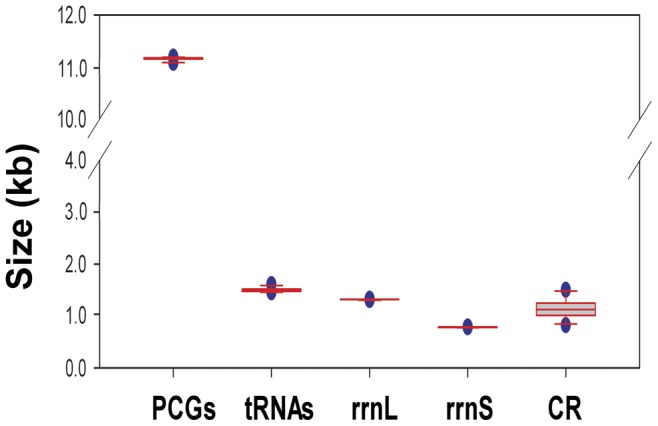
The size of PCGs, *rrnL*, *rrnS*, and CR, respectively, among sequenced Neuropterida mt genomes.

The complete mt genome of *N. punctatolosus* is a typical circular DNA molecule of 15,734 bp in length (GenBank accession number JX110703; Figure 1). The genome of this species is medium-sized when compared with genomes of other Neuropterida species, which typically range from 15,608 bp to 16,416 bp. This genome is the second largest one among the four mt genomes of Megaloptera sequenced, and relatively smaller than those of Neuroptera and Raphidioptera. Within Neuropterida mt genomes, the length variation is minimal in PCGs, tRNAs, *rrnL* and *rrnS*, but very different in the putative control region (Figure 2; Table S2). The mt genome of *N. punctatolosus* contains all 37 genes (13 PCGs, 22 tRNA genes, and 2 rRNA genes) that are typically present in metazoan mt genomes [40]. The A+T composition in this region is 91.15%, much higher than that of the coding region. Twenty-three genes were transcribed on the majority strand (J-strand), whereas 14 genes were oriented on the minority strand (N-strand). Gene overlaps were found at 14 gene junctions and involved a total of 39 bp; the longest overlap (8 bp) existed between *tRNA^Tyr^* and *cox1*. In addition to the large non-coding region, several small non-coding intergenic spacers are present in the *N. punctatolosus* mt genome and spread over nine positions, ranging in size from 1 to 14 bp (Table S3).

The gene order of the *N. punctatolosus* mt genome is the same as the ancestral gene order of *Drosophila yakuba* (Burla), which is considered to exhibit the ground pattern of insect mt genomes [41], and all gene boundaries in *D. yakuba* are conserved in the mt genome of *N. punctatolosus*. The known mt genomes of all ten Neuropterida species exhibit highly conserved gene order, with Megaloptera and Raphidioptera having the insect ancestral gene order [34], [35]. The gene order of the reported mt genomes of Neuroptera differs slightly from the putative insect ancestral gene order in the translocation of *trnC*, which is located at upstream of *trnW* but not at its traditional downstream location of *trnW*. This tRNA rearrangement might be synapomorphic for the order Neuroptera as supposed by Cameron et al. [27].

The *N. punctatolosus* mt genome contains 10 non-coding regions, extending from 1 to 1006 nucleotides. These were distributed among PCGs, tRNAs and *rrnL* and *rrnS* (Table S3). The largest non-coding region (1006 bp) is the so-called control region which was flanked by *trnS2* and *rrnL* in the *N. punctatolosus* mt genome; it was highly enriched in AT (91.15%), and has simple structure without conserved blocks and long tandem repeats.

### Base Composition and Codon Usage

**Figure 3:**
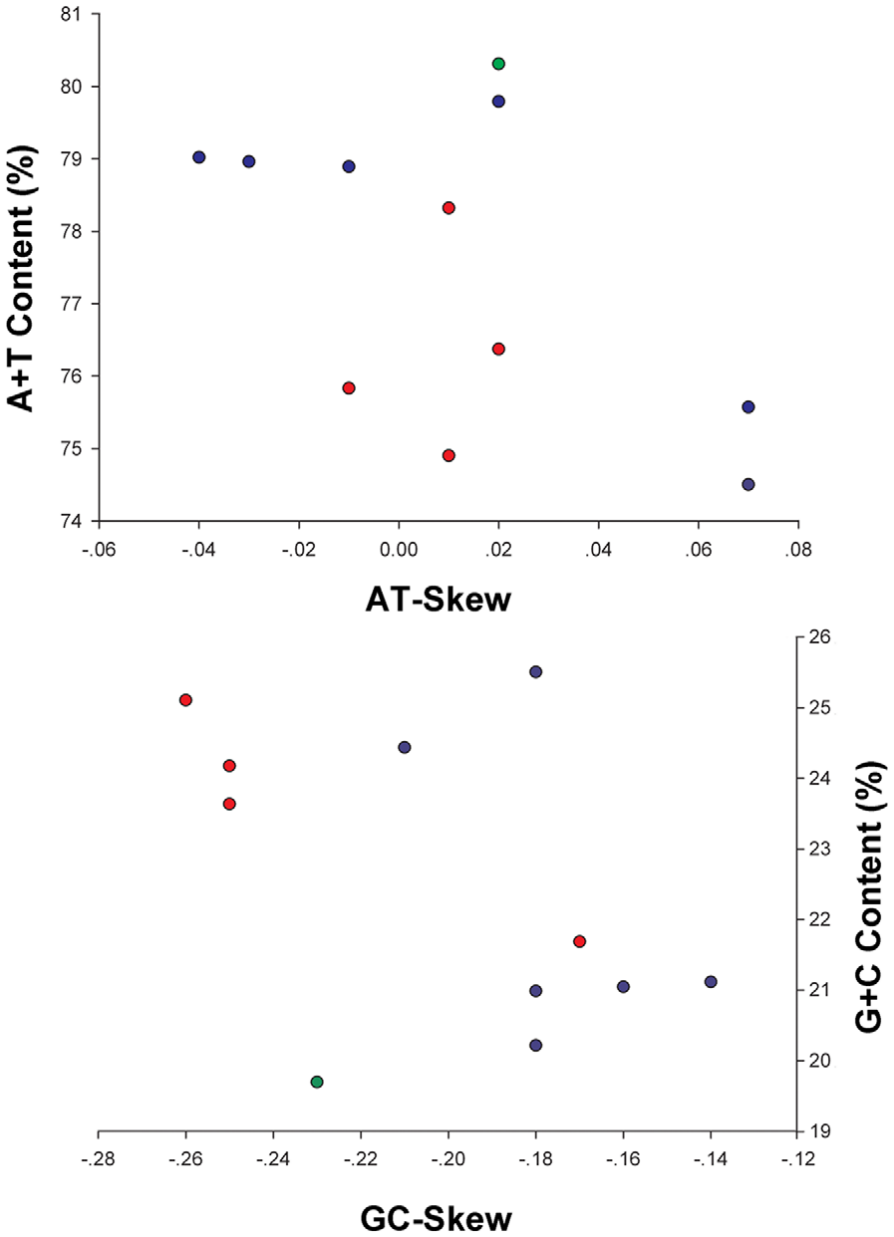
AT% vs AT-Skew and GC% vs GC-Skew in Neuropterida mt genomes. Measured in bp percentage (Y-axis) and level of nucleotide skew (X-axis). Values are calculated on full length mt genomes. Green circle, Raphidioptera; blue circle, Neuroptera; red circle, Megaloptera.

Similar to mt genome sequences of other Neuropterida species, the nucleotide composition of the *N. punctatolosus* mt genome is also biased toward A and T (A = 38.82%, T = 37.55%, G = 8.87%, C = 14.76%; Table S4). The overall AT content (76.37%) of *N. punctatolosus* is lower than the average AT content of the Neuropterida mt genomes (Table S4). The metazoan mt genomes usually present a clear strand bias in nucleotide composition [42], [43], and the strand bias can be measured as AT- and GC-skews [44]. A comparative analysis of A + T% vs AT-skew and G + C% vs GC-skew across all available mt genomes of Neuropterida is shown in Figure 3. The average AT-skew of the Neuropterida mt genomes is 0.01, ranging from −0.04 in *Apochrysa matsumurae* to 0.07 in *Libelloides macaronius* and *Ascaloptynx appendiculatus*, whereas the *N. punctatolosus* mt genome exhibits a weak AT-skew (0.02) (Table S4). The average GC-skew of Neuropterida mt genomes was −0.20, ranging from −0.26 in *Corydalus cornutus* to −0.14 in *Chrysoperla nipponensis*, and the *N. punctatolosus* mt genome exhibits a marked GC-skew (−0.25) (Table S4). AT- and GC-skews of Neuropterida mt genomes are consistent to the usual strand biases of metazoan mtDNA (positive AT-skew and negative GC-skew for the J-strand).

The 13 PCGs exhibit the canonical mitochondrial start codons for invertebrate mtDNAs [40], TTG for the *nad1* and ATN for the remaining 12 PCGs. Stop codons for the 13 PCGs were almost invariably complete TAA or incomplete TA/T. The genome-wide bias toward AT was well documented in the codon usage (Table S5). At the third codon position, A or T were overwhelmingly represented compared to G or C. The overall pattern is very similar among the mt genomes of the Neuropterida species, with similar frequency of occurrences of various codons within a single codon family. There is a strong bias toward AT-rich codons with the six most prevalent codons in *N. punctatolosus*, as in order, TTA-Leu (11.61%), ATT-Ile (9.40%), TTT-Phe (8.08%), ATA-Met (5.50%), AAT-Asn (4.23%), and TAT-Tyr (3.91%) (Table S5).

### Protein-coding Genes

The total length of all 13 PCGs was 11,167 bp, accounting for 70.97% of the entire length of *N. punctatolosus* mt genome. The overall AT content of PCGs was 74.09%, ranging from 66.84% (*cox1*) to 80.98% (*nad6*). Start and stop codons were determined based on alignments with the corresponding genes of other Megaloptera species (Table S6). Five genes (*cox2*, *atp6, cox3, nad4, cytB*) use the standard ATG start codon, three genes (*cox1, atp8, nad4l*) initiate with ATC, two genes (*nad5, nad6*) start with ATA, two genes (*nad2, nad3*) initiate with ATT, and *nad1* initiates with TTG. *Cox1* most likely starts with TTG. Ten genes employ a complete translation termination codon, either TAG (*nad3*) or TAA (*nad2, cox2, atp8, atp6, cox3, nad4L, nad6, cytB, nad1*), whereas the remaining three have incomplete stop codons, either T (*nad5*, *nad4*) or TA (*cox1*). The presence of an incomplete stop codon is common in metazoan mt genomes [40] and these truncated stop codons were presumed to be completed via post-transcriptional polyadenylation [45]. The common stop codons TAA or TAG could always overlap several nucleotides within the down-stream tRNA, which was supposed to act as “backup” to prevent translation read through if the transcripts were not properly cleaved [46]. The absence of some G + C-rich codons was found in *N. punctatolosus*: the codon AGG was not used. This result suggest that the A + T codon bias of the mt genomes affects the amino acid frequency of the encoded proteins [38].

Sequence overlaps were found between 15 neighbour genes (Table S3). In many insect mt genomes, the ATP8/ATP6 gene pairs overlap seven nucleotides (ATGATAA), and the ND4L/ND4 gene pairs overlap nucleotides (ATGTTAA). They are thought to be translated as a bicstron [47]. In the *N. punctatolosus* mt genome, the overlap nucleotides were conserved (ATGATAA for *atp8/atp6* and ATGTTAA for *nad4l/nad4*). These overlapped sequences were also observed in other three species of Megaloptera (*Corydalus cornutus*, *Protohermes concolorus*, and *Sialis hamata*) as well as two species of Neuroptera (*Polystoechotes punctatus* and *Ditaxis beseriata*). However, they were not found in the ATP8/ATP6 gene pair of Raphidioptera species *Mongoloraphidia harmandi* (Raphidioptera) and Neuroptera species *Ascaloptynx appendiculatus*.

### Transfer RNAs

**Figure 4:**
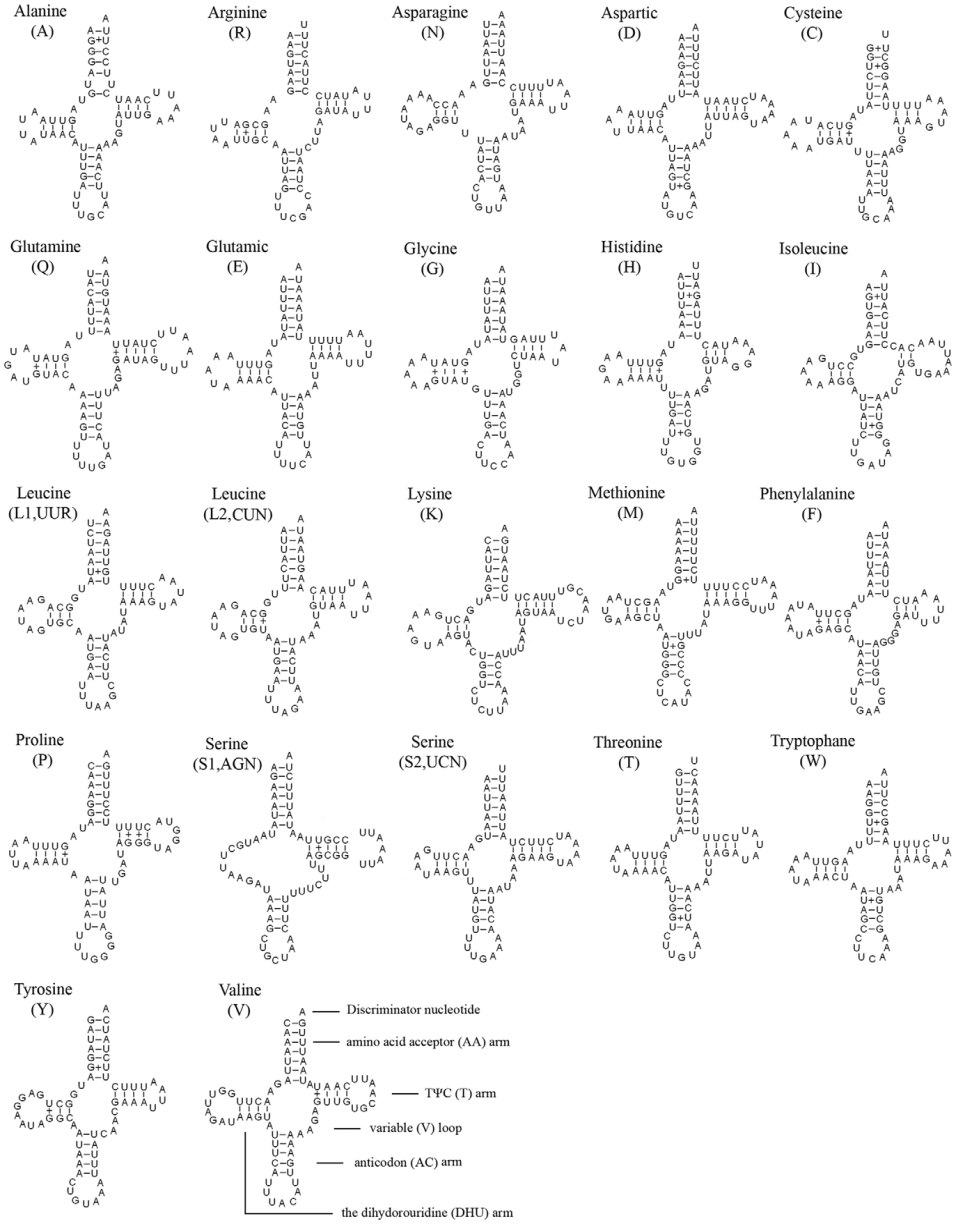
Inferred secondary structure of 22 tRNAs of *Neochauliodes punctatolosus*. The tRNAs are labelled with the abbreviations of their corresponding amino acids. Inferred Watson-Crick bonds are illustrated by lines, whereas GU bonds are illustrated by dots.

The entire complement of 22 typical tRNAs in the arthropod mt genomes was found in *N. punctatolosus* and schematic drawings of their respective secondary structures are shown in Figure 4. Most of the tRNAs could be folded as classic clover-leaf structures, with the exception of *trnS1*, in which its DHU arm simply forms a loop. This phenomenon was considered to be a typical feature of metazoan mt genomes [40] and is common in sequenced Neuropterida mt genomes. Within the 22 tRNA genes, 14 genes were encoded by the J-strand, while the remains were coded by the N-strand.

The length of tRNAs ranged from 63 to 71 bp. The aminoacyl (AA) stem (7 bp) and the AC loop (7 nucleotides) were invariable. The DHU and TΨC (T) stems are variable while the loop size (3–9 nucleotides) was more variable than the stem size (0–5 bp). The size of the anticodon (AC) stems was constantly 5 bp, except the *tRNA^Ser(AGN)^* whose AC stem size was 4 bp. Based on the secondary structure, 32 mismatched base pairs were found in *N. punctatolosus* tRNAs. Thirty of them were G–U pairs located in the AA stem (9 bp), the DHU stem (10 bp), the AC stem (6 bp), the T stem (5 bp). The remaining 2 were U-U mismatches in the AA stem of *tRNA^Ala^* and the AC stem of *tRNA^Ser(AGN)^*.

### Ribosomal RNAs

Because there is no start codon or stop codon in the rRNA genes, it is impossible to precisely infer the boundaries of the rRNAs from the DNA sequence alone, so they are assumed to extend to the boundaries of flanking genes [46], [48]. The *rrnS* was assumed to fill up the blanks between *tRNA-V* and *nad1*. For the boundary between the *rrnL* and the non-coding putative control region, alignments with homologous sequences in other Megaloptera mt genomes were applied to determine the 3′-end of the gene [27], [34], [35]. The length of *rrnL* and *rrnS* of *N. punctatolosus* was determined to be 1,318 bp and 789 bp, respectively.

**Figure 5:**
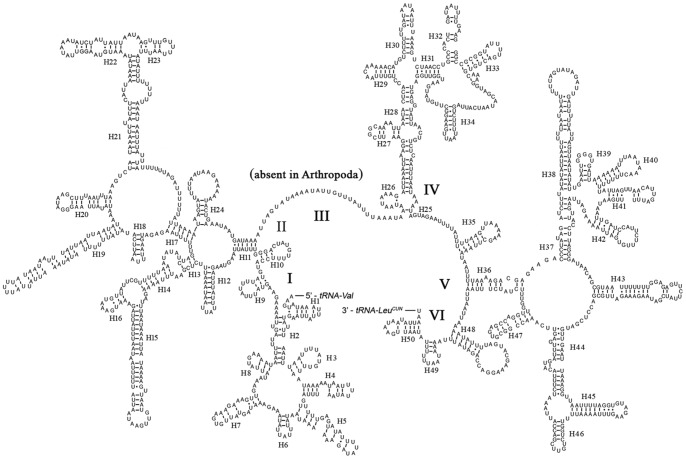
Predicted secondary structure of the *rrnL* gene in *Neochauliodes punctatolosus*. Inferred Watson-Crick bonds are illustrated by lines, GU bonds by dots.

**Figure 6:**
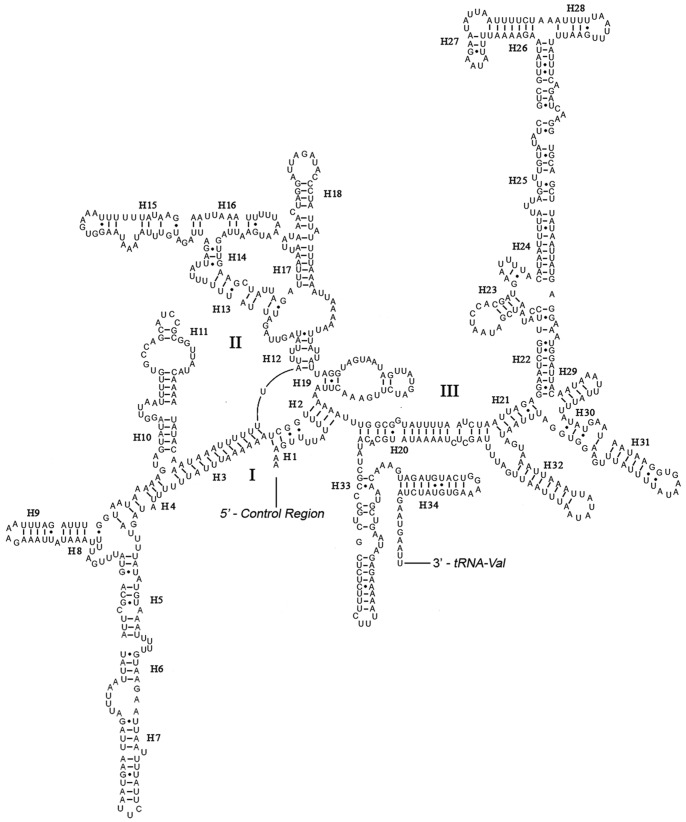
Predicted secondary structure of the *rrnS* gene in *Neochauliodes punctatolosus*. Roman numerals denote the conserved domain structure. Inferred Watson-Crick bonds are illustrated by lines, GU bonds by dots.

Both *rrnL* and *rrnS* are generally congruent with the secondary structure models proposed for other insects [39], [49]–[53]. The structure of *rrnL* of *N. punctatolosus* largely resembles previously published structures for *L. macaionius* [39], and the inferred secondary structure presents five canonical domains (I–II, IV–VI) with domain III absent, which is a typical trait in arthropods [49] (Figure 5), and includes 50 helices. The highest level of invariable positions was located on domain IV, while lowest level was on domains I–II. The *rrnS* of *N. punctatolosus* is largely in agreement with those proposed for other Holometabolan orders, including three domains and 34 helices (Figure 6).

### Phylogeny

Four datasets were used in the presented analyses, each representing different types of data partitioning and inclusion/exclusion of particular sites. There were 11608 sites in the PCG123R matrix (containing all three codon positions of PCGs, plus the two rRNA genes), 10299 sites in the PCG123 matrix (containing all three codon positions of PCGs), 8175 sites in the PCG12R matrix (containing the first and the second codon positions of PCGs, plus the two rRNA genes), and 6866 sites in the PCG12 matrix (containing the first and the second codon positions of PCGs).

**Figure 7:**
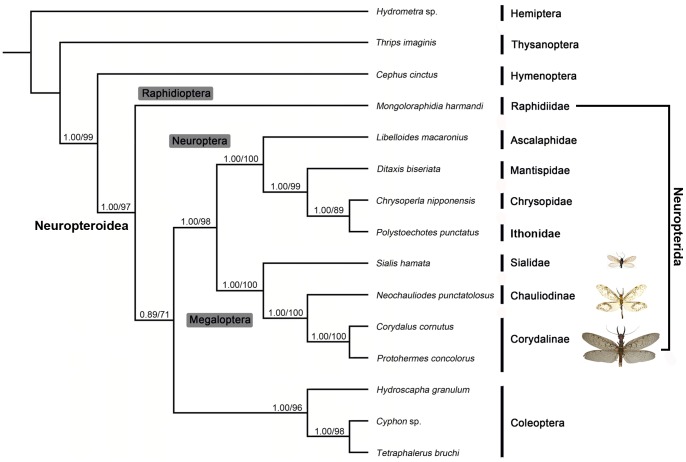
Phylogenetic relationships among the sequenced Neuropterida insects. Numbers at the nodes are Bayesian posterior probabilities (left) and ML bootstrap values (right).

The phylogenetic trees generated from Bayesian and ML inferences have similar topologies based on different datasets. The supporting values of the PCG123 matrix are higher than the other matrices. Therefore, we show the supporting values of the PCG123 matrix in Figure 7. Within Neuropterida, a close sister-relationship between Megaloptera and Neuroptera was recovered in all analyses with high statistical support, which is consistent with the result from the mt genome phylogeny of Neuropterida made by Cameron et al. [27]. However, in this paper, the single representative species of Raphidioptera was not grouped with Megaloptera and Neuroptera in Neuropterida, or even within Neuropteroidea in either Bayesian or ML analyses. This result is very surprising as the monophyly of Neuropteroidea (comprising Coleoptera, Strepsiptera, Raphidioptera, Megaloptera and Neuroptera) is now widely supported by numerous recent studies on holometabolan phylogeny based on both molecular and morphological evidence [20], [29], [30], [33], [36]. Moreover, Beutel and Friedrich [20] identify two putative synapomorphies for Neuropterida found in the larval head. This contrary result might be due to some unpredictable factor of the mt genome data when resolving such deep-level phylogenetic relationships in the Bayesian inference. Clearly increased sampling of Raphidioptera is warranted to help alleviate this perceived error in either taxon sampling or anomalous phylogeneytic signal in *Mongoloraphidia*, and thus clarify the relationship of this order with the rest of Neuropteroidea.

Megaloptera was recovered to be monophyletic in all present analyses from different datasets, which is consistent with the result from the mt genome phylogeny of Holometabola inferred by only PCGs data [32]. The limited phylogenetic utility of loci chosen as well as sparse taxon sampling for Megaloptera in the analyses made by Winterton et al. [33] was mentioned to be the main reason for the paraphyly of Megaloptera, while the mt genomic data is shown to be constantly efficient for resolving the monophyly of this order due to the large set of informative sequence data.

Within Megaloptera, the two subfamilies Corydalinae and Chauliodinae traditionally placed within the family Corydalidae were grouped as monophyletic, while the family Sialidae was recovered as sister to Corydalidae. Therefore, the relationships among the three main groups of Megaloptera herein are resolved based on mt genome data suggesting that the traditional higher classification within Megaloptera should be considered robust, whereas the assumed grouping of Sialidae + Chauliodinae [15] has been never found in any molecular phylogeny.

Besides the above findings on the phylogeny of Megaloptera, the present phylogeny also provided some new evidence for the phylogeny of Neuroptera. A three-suborder classification system of Neuroptera was proposed by Aspöck et al. [18] based on a comprehensive morphological phylogeny and the three suborders are recognized as Nevrorthiformia, Hemerobiiformia, and Myrmeleontiformia. However, this classification has never been fully recovered in any subsequent comprehensive quantitative analysis of Neuropterida phylogeny [31], [33]. In the molecular phylogeny by Haring and Aspöck [31] Myrmeleontiformia was assigned to be a sister lineage of a clade including Ithonidae + Polystoechotidae, Chrysopidae + Hemerobiidae and Mantispidae. Based on phylogenetic analysis of morphological and molecular data for both extant and extinct members of the families Ithonidae, Rapismatidae and Polystoechotidae, Winterton and Makarkin [54] showed that all members of these three families should be placed in a single family Ithonidae. In Aspöck and Aspöck [19] Myrmeleontiformia form a monophyletic group, with the ‘polystoechotid clade’ (Ithonidae) as sister group, and Mantispidae together with Berothidae are part of the dilarid clade. By contrast, a combined molecular and morphological phylogeny by Winterton et al. [33] indicated that Myrmeleontiformia form a monophyletic group with a clade including Chrysopidae and Ithonidae, while Mantispidae was outside of this group. In the present mt genomic phylogeny, Mantispidae and the sister pair of Chrysopidae + Ithonidae formed a monophyletic group, while Ascalaphidae as the representative taxon of Myrmeleontiformia was assigned to be the sister of the preceding group. This pattern is generally similar to the result obtained from Haring and Aspöck [31]. However, a more robust interfamilial phylogeny of Neuroptera can only be made in based on mt phylogenomic analysis using more comprehensive sampling of all neuropteran families.

### Divergence Time Estimation

**Figure 8:**
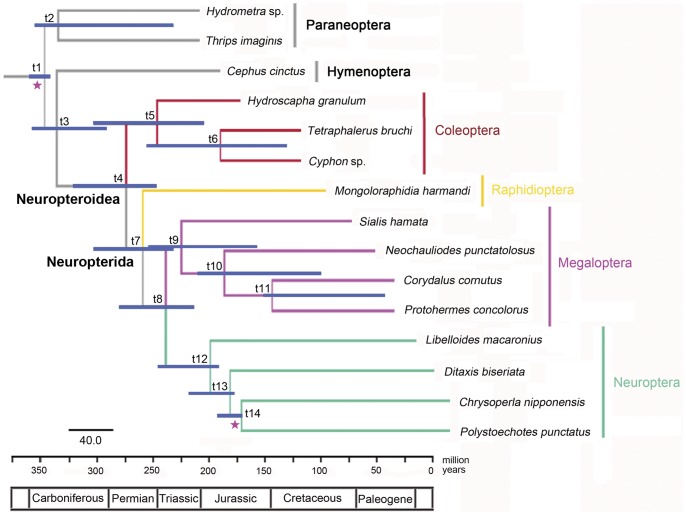
Estimated divergence times of major clades of Neuropterida. Nodes on the phylogram represent means of the probability distributions for node ages, with time intervals for 95% probability of actual age represented as blue bars. Time-scale units are in millions of years and numbers on nodes represent the estimated age for that divergence.

Hitherto, the divergence time estimation of insects based on the mt genomic data has been poorly studied. The only example refers to the phylogenetic reconstruction and divergence time estimation on Diptera based on multiple datasets, including the mt genome sequences [55]. The present analysis represents the first divergence time estimation on Neuropterida by using solely mt genomic data. The maximum clade credibility tree with median node heights and the 95% high posterior density (HPD) interval on each divergence is shown in Figure 8 and Table S7. Due to the monophyly constraint for Neuropterida, the tree topology differs from that in Figure 7, with Coleoptera being the sister clade of Neuropterida inclusive of Raphidioptera. Hymenoptera remain as sister to the rest of Holometabola, and the sister relationship between Megaloptera and Neuroptera was unchanged. Neuropterida diverged from Coleoptera in the Early Permian at 273 (95% HPD 292–357) Ma, which is generally consistent with the corresponding time estimated by Wiegmann et al. [29] based on the data from nuclear genes, but much later than the Late Carboniferous (324 Ma) estimated by Winterton et al. [33], although ranges for estimated divergences in all three analyses overlap. The earliest divergence among the orders of Neuropterida is the split between Raphidioptera and Megaloptera + Neuroptera, which was dated in the Late Permian at 258 (95% HPD 231–302) Ma. It is notable that the earliest interordinal divergence within Neuropterida was also estimated to be in the Late Permian based on the nuclear genes data by Wiegmann et al. [29] although this refers to the split between Neuroptera and Megaloptera + Raphidioptera. Nevertheless, Winterton et al. estimated that the separation of Neuroptera from Megaloptera and Raphdioptera might have happened earlier, in the Late Carboniferous at 317 Ma [33], a conclusion supported by fossil stem-group Coleoptera and Neuropterida throughout the Permian (but no evidence from the Carboniferous). The mean estimated date of divergence of Megaloptera and Neuroptera was 238 (95% HPD 214–280) Ma. This divergence time is slightly later than the Late Permian period when both earliest Megaloptera and Neuroptera arose [11]. However, considering the 95% confidence interval, the estimate also fits with the known fossil records, which indicate Megaloptera and Neuroptera originated no later than the Late Permian. Within Megaloptera, Sialidae separated from Corydalidae in the Late Triassic at 224 (95% HPD 157–254) Ma, while the earliest Sialidae is known in the Early Jurassic [14]. The mean estimated date of divergence of the lineage leading to Corydalinae and Chauliodinae was 186 (95% HPD 100–210) Ma in the Early Jurassic, which is close to but slightly earlier than the oldest fossil record of Chauliodinae in the Middle Jurassic [13]. Considering Neuroptera, all four families were estimated to be diverged by the end of the Jurassic, which corresponds with Winterton et al. [33]. For example, the mean estimated date when Ascalaphidae separated from the other three families was 199 (95% HPD 192–246) Ma in the Early Jurassic. Compared with the branching times estimated for the divergence of Myrmeleontiformia, Mantispidae + Berothidae, Chrysopidae + Hemerobiidae, and Ithonidae (all in the Triassic), the present estimation showed somewhat late divergence of the corresponding clades.

Some disadvantage of the divergence time estimation based on the mt genomic data are recognized herein. First, due to the difficulties in modeling the inherently heterogeneous patterns of mutation of various PCGs in the mt genome, the confidence intervals may not appear to have narrowed despite the use of larger mt genomic dataset than the smaller gene segments [56]. Second, the saturated nucleotide sites may underestimate the molecular distances and overestimate the branching times, especially among deep branching or early divergent taxa, when using all sites of the PCGs of the mt genome to estimate the divergence time [57]. The present estimation of interordinal and interfamilial divergences of Neuropterida also showed large confidence intervals for most nodes. However, besides the above mentioned difficulties for modeling the heterogeneous mutation of PCGs, the few nodes with constrained ages in the phylogenetic tree may also lead to such pattern of wide confidence intervals for the unconstraint nodes. Compared with the published estimated times for certain branches of Neuropterida [29], [33] based on multiple gene segments, the present estimation of the Neuropterida divergence did not show any overestimated branching times caused by the saturated nucleotide sites of the PCGs.

### Conclusion

This is the first description of the complete mt genome of a fishfly species (Megaloptera: Corydalidae: Chauliodinae). Comparative analyses suggest that the gene size, gene content, and base composition are comparatively conserved among the Neuropterida mt genomes. Most of the tRNAs can be folded as classic clover-leaf structures, with the exception of *trnS1*, in which its DHU arm simply forms a loop. The mt genomic phylogeny herein reconstructed clearly supports the monophyly of Megaloptera, the sister relationship between Megaloptera and Neuroptera, and the monophyly of Corydalidae which includes Corydalinae and Chauliodinae. The divergence time estimation based on the mt genomic data suggests that Neuropterida might be separated from Coleoptera in the Early Permian. The interordinal divergence within Neuropterida might have happened in the Late Permian, when Megaloptera and Neuroptera also arose. The Jurassic could be a significant period for the divergence of various families of Neuroptera. Future determination of the mt genomes of all Neuropterida families will draw a better resolved higher phylogeny and time-scale for this ancient but fascinate group.

## Materials and Methods

### Ethics Statement

No specific permits were required for the insect collected for this study in Yunnan. The specimen was collected by using light trap. The field studies did not involve endangered or protected species. The species in the genus of *Neochauliodes* are common in Yunnan and northern Indochina, and are not included in the “List of Protected Animals in China”.

### Samples and DNA Extraction

The *N. punctatolosus* specimen used to determine the mt DNA were collected from Mengla, Yunnan Province, China, in May 2011. After collection, it was initially preserved in 95% ethanol in the field, and transferred to −20°C for the long-term storage upon the arrival at the China Agricultural University (CAU). Total DNA was purified from muscle tissues of the thorax using TIANamp Genomic DNA Kit (TIANGEN). The quality of DNA was assessed through electrophoresis in a 1% agarose gel and staining with Gold View (nucleic acid stain replacing EB).

### PCR Amplification and Sequencing

The mt genome of *N. punctatolosus* was generated by amplification of overlapping PCR fragments (Figure 1 and Table S8). Firstly, fifteen fragments were amplified using the universal primers [58]. Then, seven specifically designed primers (Table S8) based on the known sequences were used for the secondary PCRs.

All PCRs used NEB Long Taq DNA polymerase (New England BioLabs, Ipswich, MA) under the following amplification conditions: 30 s at 95°C, 40 cycles of 10 s at 95°C, 50 s at 48–55°C, 1 kb/min at 68°C depending on the size of amplicons, and the final elongation step at 68°C for 10 min. The quality of PCR products were evaluated by agarose gel electrophoresis.

All fragments were sequenced in both directions using the BigDye Terminator Sequencing Kit (Applied Bio Systems) and the ABI 3730XL Genetic Analyzer (PE Applied Biosystems, San Francisco, CA, USA) with two vector-specific primers and internal primers for primer walking.

### Bioinformatic Analysis

The complete mt genome of *N. punctatolosus*has been deposited in GenBank under accession number JX110703. Mt DNA sequences were proof-read and aligned into contigs in BioEdit version 7.0.5.3 [59]. Sequence analysis was performed as follows. Firstly, The tRNA genes were identified by tRNAscan-SE Search Server v.1.21 [60] using invertebrate mitochondrial predictors with a COVE cutoff score of 1, or by sequence similarity to tRNAs of other Neuropterida. PCGs were identified as open reading frames corresponding to the 13 PCGs in metazoan mt genomes. The rRNA gene boundaries were interpreted as the end of a bounding tRNA gene and by alignment with other Neuropterida gene sequences. The base composition, codon usage, and nucleotide substitution were analyzed with MEGA 4.0 [61]. The GC and AT asymmetry was measured in terms of GC and AT skews using the following formulae: AT-skew  =  (A−T)/(A+T) and GC-skew  =  (G−C)/(G+C) [44]. Secondary structures of the small and large subunits of *rrnS* were inferred using models predicted for *Drosophila yakuba* [41], *Apis mellifera* [50], and *Libelloides macaronius* [39]. Stem-loops were named with Roman numbers.

### Phylogenetic Analysis

The ingroup taxa for the present phylogenetic analyses include nine species of Neuropterida, which represent three orders within the superorder and all families with available mt genomes. (Table S1). Two Paraneoptera taxa, namely *Hydrometra* sp. (Hemiptera), and *Thrips imaginis* (Thysanoptera) were selected as outgroups because of their relatively close relationships with Holometabola [11]. Three species of Coleoptera and one species of Hymenoptera were also included as outgroup taxa.

DNA alignment was inferred from the amino acid alignment of 13 PCGs using Clustal X [62]. RNA alignment was conducted by G-blocks Server (http://molevol.cmima.csic.es/castresana/Gblocks_server.html) by more stringent selection. Alignments of individual genes were then concatenated excluding the stop codons. MrBayes Version 3.1.2 [63] and a PHYML online web server [64], [65] were employed to reconstruct the phylogenetic trees. Model selection was based on Modeltest 3.7 [66] for nucleotide sequences. According to the Akaike information criterion, the GTR+I+G model was optimal for analysis with nucleotide alignments. In Bayesian inference, two simultaneous runs of 2,000,000 generations were conducted. Each set was sampled every 200 generations with a burnin of 25%. Trees inferred prior to stationarity were discarded as burnin, and the remaining trees were used to construct a 50% majority-rule consensus tree. In the ML analysis, the parameters were estimated during analysis and the nodal support values were assessed by bootstrap re-sampling (BP) [67] calculated using 100 replicates.

### Divergence Time Estimation

Divergence time estimates were calculated based on the PCG123 data matrix using the program BEAST Version 1.5.3 [68], which uses MCMC approximation to estimate the joint posterior probability of a tree topology, a set of branch lengths, rates of evolution along each branch and divergence times under a variety of substitution models, branching models and among-lineage rate-variation models. A time scale of Neuropterida was reconstructed by Winterton et al. [33] based on a phylogeny obtained from sequence data of two mitochondrial and two nuclear genes (COI +16S rDNA + CAD +18S rDNA). In order to test the utility of the mt genome data for divergence time estimation, we applied age constraints for two nodes. First, as the root of the present phylogeny representing the separation between Paraneoptera and Holometabola, we followed the recent opinion that the Holometabola originated during Early Mississippian in Carboniferous (∼355 MA) [29] and bounded the age between 360 and 340 MA, although any definitive holometabolous fossil has not been found during this period. Second, we bounded the minimum age of Ithonidae + Chrysopidae at 170 MA because the earliest fossil of this lineage was found from the Middle Jurassic [69], [70]. The input dataset comprise the sequences of 13 PCGs from 15 mt genomes. We constrained Holometabola and ingroup Neuropterida to be monophyletic respectively, and allowed all other relationships to vary. The GTR substitution model, empirical base frequencies, and speciation Yule process were applied as Tree prior. 50 million generations were run under the uncorrelated lognormal relaxed clock and sampled every 1000 generation to estimate the divergence time. Finally, we set the burnin value of 12500 under the TreeAnnotator Version 1.5.3 [68], discarding the aged samples before stationarity. The phylogenetic tree was viewed and edited by using FigTree Version 1.3.1 [71].

## Supporting Information

Table S1 **Taxa sampling in this study.**
(DOC)

Table S2 **The size of PCGs, tRNAs,** ***rrnL*** **,** ***rrnS*** **, and CR, respectively, among sequenced Neuropterida mt genomes.**
(DOC)

Table S3 **Organization of** ***Neochauliodes punctatolosus*** **mt genome.**
(DOC)

Table S4 **Base composition and strand bias in Neuropterida mt genomes.**
(DOC)

Table S5 **Codon usage of PCGs in** ***Neochauliodes punctatolosus*** **mt genome.**
(DOC)

Table S6 **Base composition and strand bias in PCGs of** ***Neochauliodes punctatolosus.***
(DOC)

Table S7 **Bayesian estimates of divergence times based on the relaxed molecular clock approach.**
(DOC)

Table S8 **Primer sequences used in this study.**
(DOC)
