# Supplementary material for: The First Mitochondrial Genome for the Fishfly Subfamily Chauliodinae and Implications for the Higher Phylogeny of Megaloptera
Source: PLoS One. 2012 Oct 9;7(10):e47302. doi: 10.1371/journal.pone.0047302 (PMC3467237; doi:10.1371/journal.pone.0047302)
Supplement: Table S1 — Taxa sampling in this study. (DOC) [file pone.0047302.s001.doc]

**Table S1.** Summary of taxonomic groups used in this study

| **Order/suborder** | **Family** | **Species** | **Accession Number** |
| --- | --- | --- | --- |
| **Hemiptera** |  |  |  |
|  | Hydrometridae | *Hydrometra* sp. | NC_012842 |
| **Thysanoptera** |  |  |  |
|  | Thripidae | *Thrips imaginis* | NC_004371 |
| **Hymenoptera** |  |  |  |
|  | Pteromalidae | *Cephus cinctus* | NC_012688 |
| **Coleoptera** |  |  |  |
|  | Scirtidae | *Cyphon* sp. | NC_011320 |
|  | Hydroscaphidae | *Hydroscapha granulum* | NC_012144 |
|  | Ommatidae | *Tetraphalerus bruchi* | NC_011328 |
| **Neuroptera** |  |  |  |
|  | Ithonidae | *Polystoechotes punctatus* | NC_011278 |
|  | Ascalaphidae | *Libelloides macaronius* | NC_015609 |
|  | Ascalaphidae | *Ascaloptynx appendiculatus* | NC_011277 |
|  | Mantispidae | *Ditaxis biseriata* | NC_013253 |
|  | Chrysopidae | *Chrysoperla nipponensis* | [NC_015093](http://www.ncbi.nlm.nih.gov/nuccore/NC_012838) |
|  | Chrysopidae | *Apochrysa matsumurae* | NC_015095 |
| **Megaloptera** |  |  |  |
|  | Sialidae | *Sialis hamata* | NC_013256 |
|  | Corydalidae | *Protohermes concolorus* | NC_011524 |
|  | Corydalidae | *Corydalus cornutus* | NC_011276 |
|  | Chauliodinae | *Neochauliodes punctatolosus* |  |
| **Raphidioptera** |  |  |  |
|  | Raphidiidae | *Mongoloraphidia harmandi* | NC_013251 |
