# Supplementary material for: The First Mitochondrial Genome for the Fishfly Subfamily Chauliodinae and Implications for the Higher Phylogeny of Megaloptera
Source: PLoS One. 2012 Oct 9;7(10):e47302. doi: 10.1371/journal.pone.0047302 (PMC3467237; doi:10.1371/journal.pone.0047302)
Supplement: Table S3 — Organization of Neochauliodes punctatolosus mt genome. (DOC) [file pone.0047302.s003.doc]

**Table S3. Organization of *Neochauliodes punctatolosus* mt genome**

| **Gene** | **Direction** | **Location (bp)** | **Size (bp)** | **Anticodon** | **Start Codon** | **Stop Codon** | **Intergenic**  **Nucleotide^*^** |
| --- | --- | --- | --- | --- | --- | --- | --- |
| *RNA^Ile^* | J | 1-64 | 64 | 29-31 GAT |  |  | 0 |
| *tRNA^Gln^* | N | 62-130 | 69 | 98-100 TTG |  |  | -3 |
| *tRNA^Met^* | J | 135-203 | 69 | 165-167 CAT |  |  | 4 |
| *ND2* | J | 204-1220 | 1017 |  | ATT | TAA | 0 |
| *tRNA^Trp^* | J | 1219-1283 | 65 | 1249-1251 TCA |  |  | -2 |
| *tRNA^Cys^* | N | 1276-1338 | 63 | 1306-1308GCA |  |  | 0 |
| *tRNA^Tyr^* | N | 1339-1409 | 65 | 1370-1372 GTA |  |  | 0 |
| *COI* | J | 1396-2936 | 1541 |  | ATC | TA-tRNA | -8 |
| *tRNA^Leu(UUR)^* | J | 2937-3000 | 64 | 2966-2968 TAA |  |  | 0 |
| *COII* | J | 3006-3686 | 681 |  | ATG | TAA | 5 |
| *tRNA^Lys^* | J | 3695-3765 | 71 | 3725-3727 CTT |  |  | 8 |
| *tRNA^Asp^* | J | 3765-3830 | 66 | 3793-3795 GTC |  |  | -1 |
| *ATP8* | J | 3831-3989 | 159 |  | ATC | TAA | 0 |
| *ATP6* | J | 3983-4660 | 678 |  | ATG | TAA | -7 |
| *COIII* | J | 4660-5448 | 789 |  | ATG | TAA | -1 |
| *tRNA^Gly^* | J | 5451-5513 | 63 | 5481-5483 TCC |  |  | 2 |
| *ND3* | J | 5513-5867 | 354 |  | ATT | TAG | 0 |
| *tRNA^Ala^* | J | 5866-5929 | 64 | 5895-5897 TGC |  |  | -2 |
| *tRNA^Arg^* | J | 5941-6003 | 63 | 5970-5972 TCG |  |  | 11 |
| *tRNA^Asn^* | J | 6003-6069 | 67 | 6034-6036 GTT |  |  | -1 |
| *tRNA^Ser(AGN)^* | J | 6069-6137 | 69 | 6095-6097 GCT |  |  | -1 |
| *tRNA^Glu^* | J | 6137-6202 | 66 | 6167-6169 TTC |  |  | -1 |
| *tRNA^Phe^* | N | 6201-6265 | 65 | 6231-6233 GAA |  |  | -2 |
| *ND5* | N | 6266-7991 | 1726 |  | ATA | T-tRNA | 0 |
| *tRNA^His^* | N | 7992-8054 | 63 | 8022-8024 GTG |  |  | 0 |
| *ND4* | N | 8054-9390 | 1336 |  | ATG | T-tRNA | 0 |
| *ND4L* | N | 9383-9674 | 291 |  | ATC | TAA | -7 |
| *tRNA^Thr^* | J | 9677-9741 | 65 | 9707-9709 TGT |  |  | 2 |
| *tRNA^Pro^* | N | 9742-9807 | 66 | 9775-9777TGG |  |  | 0 |
| *ND6* | J | 9812-10322 | 510 |  | ATA | TAA | 5 |
| *CytB* | J | 10322-11458 | 1136 |  | ATG | TAA | -1 |
| *tRNA^Ser(UCN)^* | J | 11457-11523 | 67 | 11486-11488TGA |  |  | -2 |
| *ND1* | N | 11537-12485 | 948 |  | TTG | TAA | 14 |
| *tRNA^Leu(CUN)^* | N | 12487-12550 | 64 | 12519-12521TAG |  |  | 1 |
| *lrRNA* | N | 12551-13868 | 1318 |  |  |  | 0 |
| *tRNA^Val^* | N | 13869-13939 | 71 | 13904-13906TAC |  |  | 0 |
| *srRNA* | N | 13939-14728 | 789 |  |  |  | 0 |
| *CR* |  | 14729-15734 | 1006 |  |  |  | 0 |

“*”: Negative numbers indicate that adjacent genes overlap.
