# Supplementary material for: The First Mitochondrial Genome for the Fishfly Subfamily Chauliodinae and Implications for the Higher Phylogeny of Megaloptera
Source: PLoS One. 2012 Oct 9;7(10):e47302. doi: 10.1371/journal.pone.0047302 (PMC3467237; doi:10.1371/journal.pone.0047302)
Supplement: Table S4 — Base composition and strand bias in Neuropterida mt genomes. (DOC) [file pone.0047302.s004.doc]

**Table S4. Base composition and strand bias in Neuropteran insect mt genomes**

| **Species** | **A** | **T** | **A+T** | **AT-skew** | **C** | **G** | **C+G** | **GC-skew** |
| --- | --- | --- | --- | --- | --- | --- | --- | --- |
| *Polystoechotes punctatus* | 38.34 | 40.61 | 78.96 | -0.03 | 12.22 | 8.82 | 21.04 | -0.16 |
| *Libelloides macaronius* | 39.93 | 34.57 | 74.50 | 0.07 | 15.00 | 10.50 | 25.50 | -0.18 |
| *Ascaloptynx appendiculatus* | 40.34 | 35.23 | 75.57 | 0.07 | 14.73 | 9.70 | 24.43 | -0.21 |
| *Ditaxis biseriata* | 40.52 | 39.28 | 79.79 | 0.02 | 11.92 | 8.29 | 20.21 | -0.18 |
| *Chrysoperla nipponensis* | 39.17 | 39.71 | 78.89 | -0.01 | 12.01 | 9.10 | 21.11 | -0.14 |
| *Apochrysa matsumurae* | 38.07 | 40.95 | 79.02 | -0.04 | 12.36 | 8.62 | 20.98 | -0.18 |
| *Sialis hamata* | 39.73 | 38.59 | 78.32 | 0.01 | 12.69 | 8.98 | 21.68 | -0.17 |
| *Protohermes concolorus* | 37.49 | 38.34 | 75.83 | -0.01 | 15.15 | 9.02 | 24.17 | -0.25 |
| *Corydalus cornutus* | 37.97 | 36.92 | 74.90 | 0.01 | 15.84 | 9.26 | 25.10 | -0.26 |
| *Neochauliodes punctatolosus* | 38.82 | 37.55 | 76.37 | 0.02 | 14.76 | 8.87 | 23.63 | -0.25 |
| *Mongoloraphidia harmandi* | 41.08 | 39.23 | 80.31 | 0.02 | 12.09 | 7.60 | 19.69 | -0.23 |
| Avg. | 39.23 | 38.27 | 77.50 | 0.01 | 13.52 | 8.98 | 22.50 | -0.20 |
