# Supplementary material for: The First Mitochondrial Genome for the Fishfly Subfamily Chauliodinae and Implications for the Higher Phylogeny of Megaloptera
Source: PLoS One. 2012 Oct 9;7(10):e47302. doi: 10.1371/journal.pone.0047302 (PMC3467237; doi:10.1371/journal.pone.0047302)
Supplement: Table S5 — Codon usage of PCGs in Neochauliodes punctatolosus mt genome. (DOC) [file pone.0047302.s005.doc]

**Table S5. Codon usage of protein-coding genes in the *Neochauliodes punctatolosus*** mt genome

| **AA** | **Codon*** | **n** | **%** | **RSCU** | **AA** | **Codon** | **n** | **%** | **RSCU** |
| --- | --- | --- | --- | --- | --- | --- | --- | --- | --- |
| Phe | UUU(F) | 300 | 8.08 | **1.77** | Ser | UCU(S) | 94 | 2.53 | **2.16** |
|  | UUC(F) | 39 | 1.05 | 0.23 |  | UCC(S) | 22 | 0.59 | 0.51 |
| Leu | UUA(L) | 431 | 11.61 | **4.29** |  | UCA(S) | 91 | 2.45 | 2.09 |
|  | UUG(L) | 37 | 1.00 | 0.37 |  | UCG(S) | 7 | 0.19 | 0.16 |
|  | CUU(L) | 64 | 1.72 | 0.64 | Ser(s) | AGU(S) | 34 | 0.92 | 0.78 |
|  | CUC(L) | 9 | 0.24 | 0.09 |  | AGC(S) | 12 | 0.32 | 0.28 |
|  | CUA(L) | 60 | 1.62 | 0.6 |  | AGA(S) | 88 | 2.37 | **2.02** |
|  | CUG(L) | 2 | 0.05 | 0.02 |  | AGG(S) | 0 | 0.00 | 0 |
| Ile | AUU(I) | 349 | 9.40 | **1.85** | Thr | ACU(T) | 82 | 2.21 | **1.81** |
|  | AUC(I) | 29 | 0.78 | 0.15 |  | ACC(T) | 22 | 0.59 | 0.49 |
| Met | AUA(M) | 204 | 5.50 | **1.78** |  | ACA(T) | 73 | 1.97 | 1.61 |
|  | AUG(M) | 25 | 0.67 | 0.22 |  | ACG(T) | 4 | 0.11 | 0.09 |
| Val | GUU(V) | 80 | 2.16 | **1.88** | Ala | GCU(A) | 84 | 2.26 | **1.99** |
|  | GUC(V) | 5 | 0.13 | 0.12 |  | GCC(A) | 23 | 0.62 | 0.54 |
|  | GUA(V) | 76 | 2.05 | 1.79 |  | GCA(A) | 58 | 1.56 | 1.37 |
|  | GUG(V) | 9 | 0.24 | 0.21 |  | GCG(A) | 4 | 0.11 | 0.09 |
| Tyr | UAU(Y) | 145 | 3.91 | **1.71** | Cys | UGU(C) | 36 | 0.97 | **1.89** |
|  | UAC(Y) | 25 | 0.67 | 0.29 |  | UGC(C) | 2 | 0.05 | 0.11 |
|  | UAA(*) |  | 0.00 |  | Trp | UGA(W) | 86 | 2.32 | **1.67** |
|  | UAG(*) |  | 0.00 |  |  | UGG(W) | 17 | 0.46 | 0.33 |
| His | CAU(H) | 64 | 1.72 | **1.68** | Arg | CGU(R) | 15 | 0.40 | 1.05 |
|  | CAC(H) | 12 | 0.32 | 0.32 |  | CGC(R) | 2 | 0.05 | 0.14 |
| Gln | CAA(Q) | 68 | 1.83 | **1.84** |  | CGA(R) | 37 | 1.00 | **2.6** |
|  | CAG(Q) | 6 | 0.16 | 0.16 |  | CGG(R) | 3 | 0.08 | 0.21 |
| Asn | AAU(N) | 157 | 4.23 | **1.72** | Pro | CCU(P) | 82 | 2.21 | **2.34** |
|  | AAC(N) | 26 | 0.70 | 0.28 |  | CCC(P) | 21 | 0.57 | 0.6 |
| Lys | AAA(K) | 69 | 1.86 | **1.68** |  | CCA(P) | 33 | 0.89 | 0.94 |
|  | AAG(K) | 13 | 0.35 | 0.32 |  | CCG(P) | 4 | 0.11 | 0.11 |
| Asp | GAU(D) | 61 | 1.64 | **1.79** | Gly | GGU(G) | 49 | 1.32 | 0.88 |
|  | GAC(D) | 7 | 0.19 | 0.21 |  | GGC(G) | 7 | 0.19 | 0.13 |
| Glu | GAA(E) | 72 | 1.94 | **1.82** |  | GGA(G) | 129 | 3.48 | **2.3** |
|  | GAG(E) | 7 | 0.19 | 0.18 |  | GGG(G) | 39 | 1.05 | 0.7 |

“*”: A total of 3711 codons from *Neochauliodes punctatolosus* are analyzed, excluding the start and stop codons. AA, amino acid; RSCU, Relative synonymous codon usage; n = frequency of each codon. % = n/3711.
