# Supplementary material for: The First Mitochondrial Genome for the Fishfly Subfamily Chauliodinae and Implications for the Higher Phylogeny of Megaloptera
Source: PLoS One. 2012 Oct 9;7(10):e47302. doi: 10.1371/journal.pone.0047302 (PMC3467237; doi:10.1371/journal.pone.0047302)
Supplement: Table S6 — Base composition and strand bias in PCGs of Neochauliodes punctatolosus. (DOC) [file pone.0047302.s006.doc]

**Table S6. Base composition and strand bias in PCGs of *Neochauliodes punctatolosus.***

| **Protein** | **A** | **T** | **A+T** | **AT-skew** | **C** | **G** | **C+G** | **GC-skew** |
| --- | --- | --- | --- | --- | --- | --- | --- | --- |
| *ATP6* | 33.63 | 39.09 | 72.71 | -0.08 | 16.67 | 10.62 | 27.29 | -0.22 |
| *ATP8* | 32.08 | 47.17 | 79.25 | -0.19 | 17.61 | 3.14 | 20.75 | -0.70 |
| *COI* | 28.42 | 38.42 | 66.84 | -0.15 | 18.04 | 15.12 | 33.16 | -0.09 |
| *COII* | 33.48 | 38.03 | 71.51 | -0.06 | 16.89 | 11.60 | 28.49 | -0.19 |
| *COIII* | 30.16 | 39.16 | 69.33 | -0.13 | 16.73 | 13.94 | 30.67 | -0.09 |
| *CytB* | 30.96 | 40.19 | 71.15 | -0.13 | 17.59 | 11.26 | 28.85 | -0.22 |
| *ND1* | 28.69 | 47.05 | 75.74 | -0.24 | 7.49 | 16.77 | 24.26 | 0.38 |
| *ND2* | 34.81 | 43.26 | 78.07 | -0.11 | 13.96 | 7.96 | 21.93 | -0.27 |
| *ND3* | 32.20 | 42.66 | 74.86 | -0.14 | 15.25 | 9.89 | 25.14 | -0.21 |
| *ND4* | 31.06 | 46.41 | 77.47 | -0.20 | 7.26 | 15.27 | 22.53 | 0.36 |
| *ND4L* | 27.84 | 49.83 | 77.66 | -0.28 | 5.15 | 17.18 | 22.34 | 0.54 |
| *ND5* | 31.69 | 45.42 | 77.11 | -0.18 | 7.88 | 15.01 | 22.89 | 0.31 |
| *ND6* | 38.43 | 42.55 | 80.98 | -0.05 | 13.33 | 5.69 | 19.02 | -0.40 |
| Avg. | 31.80 | 43.02 | 74.82 | -0.15 | 13.37 | 11.80 | 25.18 | -0.06 |
