# Supplementary material for: The First Mitochondrial Genome for the Fishfly Subfamily Chauliodinae and Implications for the Higher Phylogeny of Megaloptera
Source: PLoS One. 2012 Oct 9;7(10):e47302. doi: 10.1371/journal.pone.0047302 (PMC3467237; doi:10.1371/journal.pone.0047302)
Supplement: Table S7 — Bayesian estimates of divergence times based on the relaxed molecular clock approach. (DOC) [file pone.0047302.s007.doc]

**Table S7. Bayesian estimates of divergence times based on the relaxed molecular clock approach**

| **Node** | **Divergence** | **mean** | **95%HPD** |
| --- | --- | --- | --- |
| t1 | Paraneoptera-Holometabola | 346.48 | 341.61-360.00 |
| t2 | Hemiptera-Thysanoptera | 334.45 | 231.11-354.87 |
| t3 | Hymenoptera-Neuropteroidea | 335.27 | 292.02-357.36 |
| t4 | Coleoptera-Neuropterida | 273.73 | 246.71-321.09 |
| t5 | *H. granulum*-*T. bruchi*/*Cyphon* sp. | 246.18 | 204.41-302.69 |
| t6 | *T. bruchi*-*Cyphon* sp. | 189.63 | 130.58-255.53 |
| t7 | Raphidioptera-Megaloptera/Neuroptera | 258.74 | 231.45-302.41 |
| t8 | Megaloptera-Neuroptera | 238.31 | 213.65-279.71 |
| t9 | Sialidae-Corydalidae | 224.62 | 157.05-254.27 |
| t10 | Chauliodinae-Corydalidae | 186.36 | 100.16-209.57 |
| t11 | *C. cornutus*-*P. concolorus* | 143.83 | 43.97-150.85 |
| t12 | *L. macaronius*-*D. biseriata*/*C. nipponensis*/*P. punctatus* | 198.83 | 191.61-245.58 |
| t13 | *D. biseriata*-*C. nipponensis*/*P. punctatus* | 181.07 | 177.10-216.95 |
| t14 | *C. nipponensis*-*P. punctatus* | 170.68 | 170.00-191.87 |
