# Supplementary material for: The First Mitochondrial Genome for the Fishfly Subfamily Chauliodinae and Implications for the Higher Phylogeny of Megaloptera
Source: PLoS One. 2012 Oct 9;7(10):e47302. doi: 10.1371/journal.pone.0047302 (PMC3467237; doi:10.1371/journal.pone.0047302)
Supplement: Table S8 — Primer sequences used in this study. (DOC) [file pone.0047302.s008.doc]

**Table S8.** Primer sequences used in this study

| **No. fragment^*^** | **Primer ID** | **Nucleotide sequence (5’-3’)** | **Reference** |
| --- | --- | --- | --- |
| 1 | T1- J34 | GCCTGATAAAAAGGRTTAYYTTGATA | Simon *et al.*, 2006 |
|  | C1- N1738 | TTTATTCGTGGRAATGCYATRTC | Simon *et al.*, 2006 |
| 2 | C1-J1709 | AATTGGWGGWTTYGGAAAYTG | Simon *et al.*, 2006 |
|  | C1- N2776 | GGTAATCAGAGTATCGWCGNGG | Simon *et al.*, 2006 |
| 3 | C1-J2756 | ACATTTTTTCCTCAACATTT | Simon *et al.*, 2006 |
|  | C2-N3665 | CCACAAATTTCTGAACACTG | Simon *et al.*, 2006 |
| 4 | F-3665 | TGGATTTCGCTTACTAGATG | Present study |
|  | R-3790 | TGGGGATTGAGGTGTAGGAG | Present study |
| 5 | TK-J3790 | CATTAGATGACTGAAAGCAAGTA | Simon *et al.*, 2006 |
|  | A6-N4552 | ATGGTCWGCAATYATATTWGC | Simon *et al.*, 2006 |
| 6 | F-4552 | CGAGAACAAGCCATTTAACT | Present study |
|  | R-4790 | TAATAGCTACTGCTGATTCT | Present study |
| 7 | C3-J4792 | GTTGATTATAGACCWTGRCC | Simon *et al.*, 2006 |
|  | N3-N5731 | TTAGGGTCAAATCCRCAYTC | Simon *et al.*, 2006 |
| 8 | F-5731 | CAGCAGCCTGATATTGACAT | Present study |
|  | R-5747 | GGCTCCTTGATTTCATTCAT | Present study |
| 9 | N3-J5747 | CCATTTGAATGTGGRTTTGAYCC | Simon *et al.*, 2006 |
|  | TF-N6384 | TATATTTAGAGYATRAYAYTGAAG | Simon *et al.*, 2006 |
| 10 | F-6247 | CCAAAATAGAGGTTTATCAC | Present study |
|  | R-7077 | TAGGATTTCCTTTATTGGCT | Present study |
| 11 | N5-J7077 | TTAAATCCTTWGARTAAAAYCC | Simon *et al.*, 2006 |
|  | N5-N7793 | TTAGGTTGRGATGGNYTAGG | Simon *et al.*, 2006 |
| 12 | N5-J7572 | AAAGGGAATTTGAGCTCTTTTWGT | Simon *et al.*, 2006 |
|  | N4-N8727 | AAATCTTTRATTGCTTATTCWTC | Simon *et al.*, 2006 |
| 13 | N4-J8641 | CCAGAAGAACACAAACCATG | Simon *et al.*, 2006 |
|  | N4L-N9629 | GTTTGTGAGGGTGCAATAGG | Simon *et al.*, 2006 |
| 14 | F-9629 | CCATTCTTATAAACACTTTC | Present study |
|  | R-9648 | GATAGGGTAGGTACTTTTCC | Present study |
| 15 | N4L-J9648 | TCCCAACACACCTTCACAAAC | Simon *et al.*, 2006 |
|  | CB- N11010 | TATCAACAGCAAATCCTCCTCA | Simon *et al.*, 2006 |
| 16 | CB-J11335 | CATATTCAACCWGAATGRTA | Simon *et al.*, 2006 |
|  | N1-N12067 | AATCGWACTCCWTTTGATTTTGC | Simon *et al.*, 2006 |
| 17 | N1-J11876 | CGAGGTAAAGTMCCWTTTGATTTTGC | Simon *et al.*, 2006 |
|  | N1-N12595 | GTWGCTTTTTTAACTTTATTRGARCG | Simon *et al.*, 2006 |
| 18 | N1-J12261 | AACTTCATAAGAAATAGTYTGRGC | Simon *et al.*, 2006 |
|  | LR-N13000 | TTACCTAGGGATAACAGCGTAA | Simon *et al.*, 2006 |
| 19 | F-13000 | ATTACACTATTCTGCCAAAG | Present study |
|  | R-13342 | TTAAGGGATAAGCTTTAATT | Present study |
| 20 | LR-J13342 | CCTTAGCACAGTTAAAATACTGC | Simon *et al.*, 2006 |
|  | LR-N14220 | TTATGCACATATCGCCCGTC | Simon *et al.*, 2006 |
| 21 | LR-J14197 | GTAAAYCTACTTTGTTACGACTT | Simon *et al.*, 2006 |
|  | SR-N14745 | GTGCCAGCAAYCGCGGTTATAC | Simon *et al.*, 2006 |
| 22 | F-14745 | GATTATGGAACAGATTCCTC | Present study |
|  | R-34 | GGTATGAGCCCATTAGCTTC | Present study |

“*”: The orientation is shown in Figure 1.
